# Acute Safety and Efficacy of Fluoroless Cryoballoon Ablation for Atrial Fibrillation

**DOI:** 10.19102/icrm.2021.120205

**Published:** 2021-02-15

**Authors:** Daniel Alyesh, Ganesh Venkataraman, Austin Stucky, John Joyner, William Choe, Sri Sundaram

**Affiliations:** ^1^Cardiac Electrophysiology, South Denver Cardiology Associates, Littleton, CO, USA; ^2^Colorado Heart and Vascular, Lakewood, CO, USA; ^3^Abbott, Chicago, IL, USA; ^4^Medtronic, Minneapolis, MN, USA

**Keywords:** Ablation, atrial fibrillation, cryoballoon, fluoroless

## Abstract

Pulmonary vein isolation (PVI) is widely used for the ablation of atrial fibrillation, with prior reports suggesting good efficacy. Due to the widespread use of three-dimensional electroanatomic mapping systems and advances in intracardiac echocardiography, fluoroless ablation has been made possible. Fluoroless ablation with a cryoballoon (CB), however, has not been widely performed because of the need to prove occlusion of the vein with contrast dye and fluoroscopy. The objective of this study is to show that CB ablation can be performed safely and effectively without fluoroscopy. A dual-center, case–control study was performed of patients undergoing CB PVI with a fluoroless approach and a control group with traditional fluoroscopic techniques. The absence of color-flow Doppler signals around the periphery of the CB on intracardiac echocardiography and an increase in mean pressure by 5 mmHg, loss of the A-wave, and an increase in the V-wave as measured with continuous-wave pressure monitoring were adopted as indicators of vein occlusion in the absence of fluoroscopy. Temperature at 30 seconds, minimum temperature, time to isolation, procedure length, and complications were evaluated. During the study period of November 15, 2018 to November 15, 2019, a total of 100 patients underwent CB PVI at the participating centers. A total of 50 patients were enrolled in the fluoroless arm [35 men (70%), mean age: 64.9 ± 12 years, mean left atrium size: 44.2 ± 16 mL/m^2^, left ventricular ejection fraction: 61% ± 5%], while 50 patients were enrolled in the control arm with similar characteristics. Four hundred forty-one 441 PVs were evaluated in the study cohort compared to 339 PVs in the control arm. When comparing fluoroless and traditional techniques, the mean temperature at 30 seconds was −31.7°C ± 6°C versus −32.8°C ± 5°C (p = 0.037), the minimum temperature was −47.4°C ± 6°C versus −47.7°C ± 9°C (p = 0.677), the time to isolation was 56.8 ± 28 seconds versus 74.8 ± 45 seconds (p = 0.212), and the procedure time was 102.2 ± 27.3 seconds versus 104.5 ± 16.9 seconds (p = 0.6436). Ultimately, **t**his proof-of-concept study revealed that fluoroless ablation can be performed with success and efficiency outcomes similar to those of a traditional ablation approach. This suggests that the ablation of atrial fibrillation with CB can be performed safely and effectively without the use of fluoroscopy by experienced operators.

## Introduction

Pulmonary vein isolation (PVI) with either radiofrequency (RF) energy or cryoballoon (CB) technology is a well-established ablative approach for the management of atrial fibrillation (AF), with good reported efficacy rates. With widespread use of three-dimensional (3D) electroanatomic mapping systems and advances with intracardiac echocardiography (ICE), fluoroless ablation has become possible. This is desirable given the benefits of avoiding ionizing radiation as well as due to the potential orthopedic and other benefits operators and staff might experience. Fluoroless ablation using an RF energy source has been well-described, with a similar safety profile and outcomes as those of standard approaches.^1–5^ Fluoroless ablation with CB, however, has not been widely performed because of the perceived need to prove circumferential occlusion of the PV ostium with fluoroscopic evaluation of contrast dye injections into the targeted vein.

The reduction of fluoroscopy time during CB ablation has been achieved with the use of alternative imaging and hemodynamic modalities. Transesophageal echocardiography (TEE) has been shown to be an effective tool for reducing fluoroscopy exposure.^6–8^ Similarly, recent reports have demonstrated that, with the use of ICE, fluoroscopy can be reduced.^9^ A reduction in fluoroscopy has also been achieved with the use of continuous-wave pressure monitoring (CWPM) as a hemodynamic measure of vein occlusion.^10–13^ The aim of this proof-of-concept study was to show that, by employing imaging with ICE and hemodynamic measures with CWPM, fluoroscopy use during CB procedures can be safely and effectively eliminated. We present the first proof-of-concept, case–control study comparing the use of traditional fluoroscopic methods of CB ablation with a completely fluoroless approach.

## Methods

### Study participants

Study enrollment occurred from November 15, 2018 to November 15, 2019 at two high-volume AF ablation centers. All patients enrolled in the trial had drug-refractory, symptomatic paroxysmal AF or recent-onset (< 6 months) persistent AF. All patients received general anesthesia during their treatment and were monitored overnight and discharged home the next day.

Our study was a retrospective analysis of cases performed by four operators. All operators were experienced and had performed more than 100 CB ablation procedures to date. Abbott (Chicago, IL, USA) and Medtronic (Minneapolis, MN, USA) employees assisted with the data collection but did not participate in the data analysis nor the writing of this manuscript. This study was approved by the local institutional review board, who waived the need for participant consent due to the retrospective nature of this investigation.

### Ablation procedure

All patients were anticoagulated for one month prior to their procedure either with a direct oral anticoagulant or with warfarin to achieve an international normalized ratio range of 2.0 to 3.0. For patients treated with the former, their dose was held the morning of the procedure, while those on warfarin maintained their dosing schedule. Antiarrhythmic medication management prior to ablation was left to the discretion of the operator. All procedures were performed with general anesthesia. A preoperative computed tomography (CT) scan to evaluate the PV anatomy and TEE imaging to evaluate for left atrial appendage thrombus were conducted at the discretion of the operator.

**Figure 1:**
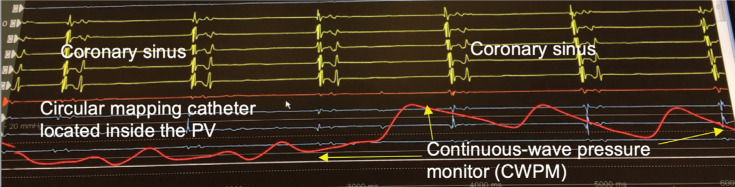
Change in CWPM with occlusion of the PV. Note the loss of the A-wave, increase in V-wave, and overall increase in the mean pressure. CWPM: continuous-wave pressure monitoring; PV: pulmonary vein.

Vascular access was obtained under ultrasound guidance. Heparin was infused to maintain an activated clotting time goal of 300 to 350 seconds. An ICE catheter (ViewFlex Xtra ICE catheter; Abbott, Chicago, IL, USA or Accuson, AcuNav ultrasound catheter; Biosense Webster, Diamond Bar, CA, USA) was advanced into the right atrium and used for the visualization of all cardiac chambers throughout each case. One transseptal puncture was performed with a fixed sheath (SL-1; Abbott) and a transseptal needle (BRK-1; Abbott) using ICE or fluoroscopic guidance. A guidewire was advanced through the SL-1 and then exchanged for a steerable sheath (Flexcath Advance Steerable Sheath; Medtronic). Using an impedance-based electroanatomic 3D mapping system (Ensite Precision™; Abbott) geometry of the left atrium (LA) and PVs were acquired using a circular mapping catheter (Reflexion Spiral; Abbott). If CT was performed, the geometry was displayed along with the anatomy from the CT scan that was acquired prior to ablation. An esophageal temperature probe (Level 1 Acoustascope 12-French; Smiths Medical ASD, Inc., St. Paul, MN, USA) was placed and monitored for changes. The temperature probe was visualized with ICE or fluoroscopy and was moved inferiorly and superiorly in accordance with the location of the CB. In the fluoroless cohort, the ICE catheter was rotated to show the posterior aspects of the LA. With this view, the esophagus can be visualized and the temperature probe can be visualized as it is placed at the level of the PVs. Ablation lesions were stopped at an esophageal temperature threshold of 25°C. Ablation was performed using an Arctic Front Cryocath Advance and Advance Pro (Medtronic) 28-mm diameter balloon in all cases. The balloon has an inner lumen guidewire that is usually used to inject contrast fluid through the distal tip of the balloon. For the nonfluoroscopic cases, the inner lumen was connected to a pressure transducer to record CWPM. A multipolar mapping catheter (Achieve; Medtronic) was also placed through the inner lumen and used for the detection of PV signals as well as to assess the entrance and exit block at the end of the case. The vein was considered occluded in the fluoroscopic arm if there was no retrograde flow into the LA with the injection of contrast through the distal port. In the nonfluoroscopic arm, the vein was considered occluded if there was a change in the mean CWPM of 5 mmHg or greater in patients in AF and an increase in the V-wave amplitude. For patients in sinus rhythm, the vein was considered occluded if there was a change in CWPM of 5 mmHg or greater and an increase in the V-wave and decrease in the A-wave to achieve a pattern consistent with the transcapillary pulmonary arterial pressure **(Figure 1)**. In addition, for the vein to be considered occluded, an absence of high-velocity Doppler color flow around the periphery of the CB on interrogation with ICE was necessary. The ICE catheter was manipulated to show all aspects of the PV. If leakage was detected, the CB, sheath, or both were adjusted until the CWPM and Doppler color-flow interrogation showed no evidence of flow.

**Figure 2:**
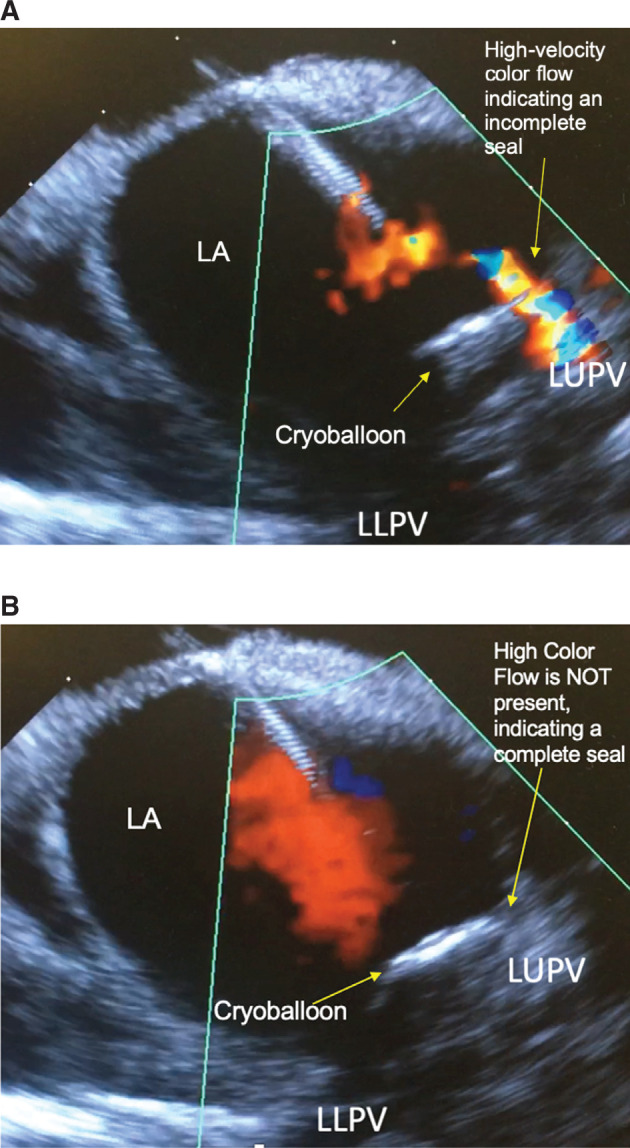
A: ICE image of the LA. The CB partially occluded the flow around the periphery of the vein. Note the presence of Doppler color flow around the right side of the CB. B: ICE image of the LA. CB fully occluded flow around the periphery of the vein. Note the absence of Doppler color flow around the right side of the balloon. CB: cryoballoon; ICE: intracardiac echo; LA: left atrium; LLPV: left lower pulmonary vein; LUPV: left upper pulmonary vein.

In rare instances, complete occlusion could not be achieved with a single CB application. These veins were segmentally ablated to achieve isolation by focusing contact on different aspects of the PV **(Figures 2A and 2B)**. Each CB freeze was applied for either 180 seconds total or until 120 seconds beyond isolation. If isolation was not achieved or less than 120 seconds of ablation were delivered following isolation, a second ablation lesion was delivered for either 120 seconds or 180 seconds, at the operator’s discretion. During ablation of the right-sided veins, the phrenic nerve was paced with a catheter in the superior vena cava or with compound motor action potential (CMAP), at the discretion of the operator. Phrenic nerve injury was particularly monitored. The location of the CB was monitored on ICE to make sure that it was ostial and not too deep in the PV. In addition, CMAP and/or palpation of the right hemidiaphragm was monitored throughout. At the completion of ablation of all the PVs, cardioversion was performed for patients that remained in AF. Afterward, all of the veins were reevaluated for the presence of PV potentials and with pacing maneuvers that showed the entrance and exit block. If necessary, further ablation lesions were delivered to achieve isolation and block. The temperature at 30 seconds, minimum temperature, time to isolation, thaw time to 20°C, and procedural complications were recorded.

### Statistical analysis

All values are reported as mean ± standard deviation. Study and control groups were compared using parametric (difference in means) and nonparametric (Wilcoxon rank-sum test) tests for continuous variables as appropriate and Fisher’s exact test for categorical data (SAS version 9.4; SAS Institute, Cary, NC, USA). A p-value of less than 0.05 was considered to be statistically significant.

## Results

### Total study cohort

During the study period of November 15, 2018 to November 15, 2019, a total of 100 patients underwent CB PVI at participating centers. Fifty consecutive patients who underwent fluoroless ablation were compared to 50 matched controls. A retrospective analysis was performed of the two cohorts. The baseline characteristics are summarized in **Table 1**. The cohort’s average age was 64.9 ± 11.5 years and 62% of participants were male. Moreover, 77% of patients had paroxysmal AF and 23% had early persistent AF. Overall, the average LA volume was 40.6 ± 14 mL/m^2^, the left ventricular ejection fraction (LVEF) was 60.5% ± 6.2%, and the CHA_2_DS_2_-VASc score was 1.82 ± 1.4 points.

### Fluoroless cohort

Fifty consecutive patients were enrolled in the fluoroless CB arm. Fluoroscopy was not used for any portion of the ablation procedure, including during transseptal access to the LA. Of the study participants, 35 were male (70%), with an age of 64.9 ± 8.1 years, LA volume of 44.2 ± 17 mL/m^2^, LVEF of 61.2% ± 5.5%, and CHA_2_DS_2_-VASC score of 1.51 ± 1.4 points recorded **(Table 1)**. A total of 198 PVs were treated with 444 CB applications. There were two patients with left common ostia that were segmentally ablated. The treated veins were divided into four categories: those with both ICE and the CWPM evidence of occlusion (n = 213; category 1), those with only CWPM evidence (n = 121; category 2), those with only ICE evidence (n = 13; category 3), and those without evidence of occlusion with either modality (n = 97; category 4). The veins in categories 1 through 3 did show isolation, while none of the veins in category 4 were isolated. Measures of CB lesion efficacy are summarized in **Table 2**. Importantly, in terms of performance, category 1 veins were the strongest, followed by category 2 and category 3 veins. Veins in category 4 were segmentally ablated and thus had inferior metrics.

### Control cohort

Fifty consecutive matched patients were also enrolled in the traditional CB arm. Among these participants, 27 were male (54%), with an age of 64.9 ± 10.6 years, LA volume of 37.0 ± 12 mL/m^2^, LVEF of 59.7% ± 6.7%, and CHA_2_DS_2_VASc score of 2.1 ± 1.3 points recorded **(Table 1)**. The veins in this cohort were divided into those with fluoroscopic evidence of occlusion (n = 187; category 1) and those without (n = 152; category 2). Veins in category 2 were segmentally ablated. Measures of CB lesion efficacy are summarized in **Table 3**. Veins with fluoroscopic evidence of occlusion performed superiorly in all of the following metrics: temperature at 30 seconds (p < 0.0001), minimum temperature (p < 0.0001), and thaw time (p < 0.0001).

### Comparing the fluoroless and control cohorts

**Figure 3:**
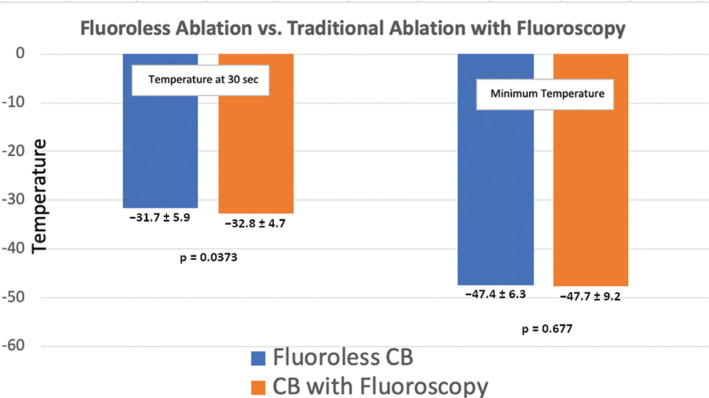
Comparison of fluoroless ablation (category 1) and traditional ablation with fluoroscopy (category 1). The results show that the temperature at 30 seconds was lower when using the traditional method but the overall absolute temperature was similar.

**Figure 4:**
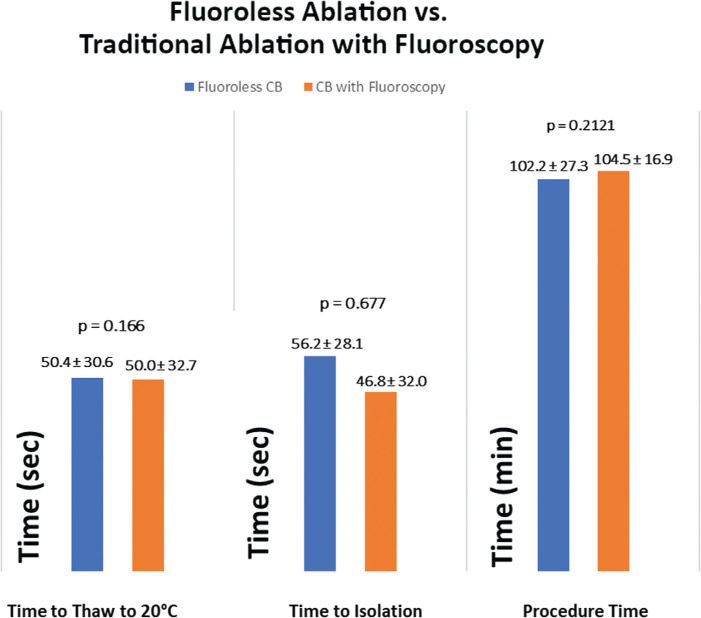
Comparison of fluoroless ablation (category 1) and traditional ablation with fluoroscopy (category 1). The results show that the thaw time to 20°C, time to vein isolation, and procedure time were similar.

When comparing patients with complete fluoroless evidence of occlusion (fluoroless category 1) and those with fluoroscopic evidence of occlusion (control category 1), the mean temperature at 30 seconds was slightly lower in the traditional approach at −31.7°C ± 5.8°C versus −32.8°C ± 4.7°C (p = 0.03), while the maximum temperature (p = 0.6755) and the time to isolation (p = 0.2121) were not different **(Figures 3 and 4)**. The full details of these comparisons are summarized in **Table 4**. When comparing veins without fluoroless ablation (category 4) and traditional evidence of occlusion (category 2), there also was no difference in the mean temperature at 30 seconds (p = 0.65), maximum temperature (p = 0.8762), or thaw time (p = 0.116) revealed.

When comparing veins with only one of the two nonfluoroscopic measures of occlusion—either ICE or CPWM alone (fluoroless categories 2 and 3)—to controls with clear evidence of occlusion (control category 1), the fluoroless cohort performed inferiorly. When comparing between fluoroless category 2 and category 1 of the control group, the temperature at 30 seconds was −30.0°C ± 4.0°C versus −32.8°C ± 4.7°C (p ≤ 0.0001), the minimum temperature was 41.9°C ± 5.9°C versus 47.7°C ± 9.1°C (p ≤ 0.0001), and the time to isolation was 56.3 ± 18.7 seconds versus 50.3 ± 32.7 seconds (p = 0.5707). In comparing fluoroless category 3 with control category 1, the temperature at 30 seconds was −28.8°C ± 2.8°C (p = 0.0007), the minimum temperature was 40.3°C ± 1.7°C (p ≤ 0.0001), and the time to isolation was 124 ± 44 seconds (p = 0.0019).

Overall, there were more CB applications delivered (444 vs. 339 applications) with the fluoroless approach, although the procedure time was not different between groups (102.2 ± 27.3 vs. 104.5 ± 16.9 seconds; p = 0.6436). As data emerged demonstrating that dosing with single lesions was durable, fewer lesions were delivered in the control arm.^14^ Four of 198 (2.0%) PVs in the fluoroless arm and two of 200 (1.0%) PVs in the control arm could not be isolated with CB alone and required a touch-up with RF energy. Complications such as phrenic nerve injury, pericardial effusion, tamponade, stroke, and death were monitored but did not occur in either study group.

## Discussion

This is a first-of-its-kind, proof-of-concept study of fluoroless CB AF ablation that has important implications. We present an approach that employs both imaging and hemodynamic measures as a viable alternative to the fluoroscopic evaluation of PV occlusion with CB. Our fluoroless technique performed similarly in terms of many of the traditional measures of CB lesion quality and was equivalent in both safety and procedure time. Importantly, the tools of ICE and CWPM are widely available in most laboratories and, thus, this technique is readily accessible for many operators. To our knowledge, this is the first study to combine ICE color Doppler and CWPM as a technique to completely eliminate fluoroscopy. There was no fluoroscopy used for any portion of the procedure in the fluoroless arm. This study constitutes an important step forward in a movement to eliminate fluoroscopy in electrophysiology (EP) procedures that has key implications for procedural radiation and orthopedic complications among operators and staff.

### Lesion quality and durability

The achievement of high-quality and durable lesions is very important in any technique for AF ablation. In the case of CB ablation, it important to achieve certain critical endpoints to ensure that a patient’s PVs are durably isolated. For this reason, we elected to include many of the most important metrics in our study. These metrics include the absolute nadir of temperature during the cryoablation, 30-second temperature, time to isolation, and thaw time.

In our study, the fluoroscopy-free cohort had a slightly higher 30-second temperature but similar values of maximum temperature, thaw time, and time to isolation. Importantly, although the fluoroless cohort performed slightly worse with respect to the 30-second temperature metric, this has not been shown to be a reliable indicator of durable long-term lesions, while the other markers are better correlated with long-term lesion durability^15^ **(Figure 3)**. With regard to the importance of absolute temperature during a freeze application, temperatures below −20°C to −50°C have also been shown to have an increased incidence of durable isolation.^16,17^ The absolute temperatures achieved in this study were similar in both arms, indicating that there is likely a similar degree of contact with the CB and the PV ostial tissue achieved with nonfluoroscopic techniques. A highly important CB ablation target is the time to isolation or effect, which, when achieved, indicates durable isolation.^14^ There was no statistically significant difference in the time to effect in patients who had either nonfluoroscopic or traditional evidence of vein occlusion (56.26 ± 28.1 vs. 46.6 ± 32.6 seconds; p = 0.212) **(Figure 4)**. Another important aspect of this finding was that nonfluoroscopic techniques also achieved isolation within the traditional time frame of one minute on average.

It is important to point out that, based upon our study results, attaining evidence of occlusion via both ICE and CPWM is important for confirming durable lesions. When comparing veins with either ICE or CPWM evidence of occlusion alone with those with fluoroscopic evidence of occlusion, the fluoroless ablation cohort performed inferiorly in all metrics. Based on these results, we must conclude that, without both markers, a successful fluoroscopy-free procedure will not be achieved.

### Adaptability to other operators

It is important to note that procedure time was similar between the two groups and major complications were absent. This suggests that, with experienced operators, a transition to a fluoroless approach is safe, efficacious, and possible with most laboratories’ existing equipment. While this concept was not studied, we would suggest that transitioning to a fluoroless approach may be undertaken by experienced electrophysiologists who have completed at least 100 CB ablation procedures. While this number is not rigorously studied, it is a reasonable experiential estimate.

### The impetus for transitioning to fluoroless procedures

There are many reasons supporting why EP procedures should move to reduce and eliminate the use of fluoroscopy. According to the “as low as reasonably achievable” principle, radiation exposure during medical procedures should be limited as there is no known safe procedural dose of radiation.^18^ EP laboratory staff and operators, in comparison with the general population, exhibit two times the risk of skin lesions, three times the risk of cancer, 6.3 times the risk of cataracts, and 7.1 times the risk of orthopedic injuries.^19–21^ In addition, EP patients are exposed to an average of approximately 15 millisieverts during an average AF ablation.^12^ This is an equivalent radiation exposure of 750 chest X-rays. Given the increased risks associated with ionizing radiation, the need for fluoroscopy reduction in cardiac EP procedures is clearly apparent.

With the introduction of CB technology, however, the traditional dogma is that the use of fluoroscopy has remained necessary to assess for the complete occlusion of the PV—a concept we would like to challenge with our study findings. With the improvement in resolution with 3D anatomic mapping systems, ICE imaging, and PV hemodynamic monitoring, fluoroscopy can be safely and effectively eliminated as evidenced by our proof-of-concept study. As EP procedures are appropriately being expanded and offered at higher volumes, the potential burden of fluoroscopy of these procedures similarly increases. In our view, the concepts outlined in our study are critical to improving how the EP community offers these important procedures.

### Limitations

This was a retrospective study of cases performed at two high-volume AF ablation centers and serves as a proof-of-concept study. More multicenter prospective and randomized data are needed with long-term clinical follow-up before this method can be widely adopted. It is important to note that the procedures in this study were performed by experienced operators. Fluoroscopy-free procedures require familiarity with the equipment and the ablation technique and further investigations are needed to understand the optimal time and process an electrophysiologist should take to transition to fluoroless CB ablation. For these reasons, we must conclude that this technique is not able to be used by inexperienced operators. In addition, the fluoroless procedure relies predominantly on the use of an ICE catheter. While the use of ICE and 3D mapping is the standard of care in the United States, this is not the case in other countries. The additional expense associated with using an ICE catheter or 3D mapping system may also be a barrier to widespread adoption of a fluoroless approach. Finally, an additional minor limitation is that the two study cohorts were slightly different, in that the fluoroless cohort had a larger LA volume and the control cohort had an elevated CHA_2_DS_2_-VASc score; however, this minor limitation likely did not contribute to any significant variation in the outcomes of this study.

## Conclusion

In comparison with traditional CB ablation with fluoroscopy, this proof-of-concept study reveals fluoroless CB ablation can be performed similarly to the traditional approach with regard to the metrics of procedure success and efficiency. Safety was comparable between the ablation approaches as there were no major complications in either arm of the study. This suggests that the ablation of AF using CB technology can be conducted safely and effectively without the use of fluoroscopy by experienced operators.

## Figures and Tables

**Table 1:** Demographics

	Fluoroless Cohort	Control Cohort	p-value
N	50	50	
Male sex	35 (70%)	27 (54%)	0.8939
Age	64.9 ± 12.0 years	64.9 ± 10.6 years	0.993
LA volume (ms/m^2^)	44.2 ± 16	37.0 ± 12	0.0236
LVEF	61.2% ± 5.6%	59.7% ± 6.7%	0.1174
CHA_2_DS_2_-VASc score	1.54 ± 1.4 points	2.1 ± 1.3 points	0.0371

LA: left atrium; LVEF; left ventricular ejection fraction.

**Table 2:** Fluoroless Cohort

	Category 1	Category 2	Category 3	Category 4
Category details	Occlusion with CWPM Occlusion with ICE	Occlusion with CWPM No occlusion with ICE	No occlusion with CWPM Occlusion with ICE	No occlusion with CWPM No occlusion with ICE
No. of freezes	213	121	13	97
Temperature at 30 seconds	−31.7°C ± 5.9°C Category 1 vs. 2: p = 0.0038 Category 1 vs. 3: p = 0.0378 Category 1 vs. 4: p ≤ 0.001	−29.9°C ± 4.0°C Category 2 vs. 3: p = 0.164 Category 2 vs. 4: p = 0.025	−28.83°C ± 2.8°C Category 3 vs. 4: p = 0.7142	−27.78°C ± 6.6°C
Minimum temperature	−47.4°C ± 6.3°C Category 1 vs. 2: p ≤ 0.0001 Category 1 vs. 3: p ≤ 0.0001 Category 1 vs. 4: p ≤ 0.0001	−41.9°C ± 5.9°C Category 2 vs. 3: p ≤ 0.0001 Category 2 vs. 4: p = 0.197	−40.33°C ± 4.7°C Category 3 vs. 4: p = 0.412	−38.9°C ± 4.6°C
Thaw time to 20°C	50.4 ± 3.6 seconds Category 1 vs. 2: p = 0.0069 Category 1 vs. 3: p = 0.0278 Category 1 vs. 4: p = 0.1189	28.3 ± 13.6 seconds Category 2 vs. 3: p = 0.0206 Category 2 vs. 4: p = 0.4978	12.8 ± 6.3 seconds Category 3 vs. 4: p = 0.0475	31.28 ± 17.9 seconds
Time to isolation	56.2 ± 28.1 seconds Category 1 vs. 2: p = 0.9504 Category 1 vs. 3: p = 0.0042 Category 1 vs. 4: p = N/A	56.25 ± 18.87 seconds Category 2 vs. 3: p = 0.05 Category 2 vs. 4: p = N/A	124 ± 43.0 seconds Category 3 vs. 4: p = N/A	N/A (isolation not achieved)

CWPM: continuous-wave pressure monitoring; ICE: intracardiac echo; N/A: not applicable.

**Table 3:** Fluoroscopy Cohort

	Category 1	Category 2	p-value
Category details	Occlusion with visual Inspection	No occlusion with visual inspection (segmental ablation was performed)	
No. of freezes	187	152	
Temp at 30 seconds	32.8°C ± 4.7°C	28.0°C ± 4.8°C	< 0.0001
Minimum temperature	47.7°C ± 9.2°C	39.63°C ± 6.6°C	< 0.0001
Thaw time to 20°C	50.03 ± 32.7 seconds	26.0 ± 16.8 seconds	< 0.0001
Time to isolation	46.79 ± 32.0 seconds	N/A (isolation not achieved)	N/A

**Table 4:** Fluoroless Cohort with CWPM and ICE Showing Occlusion (Category 1) Versus Traditional Ablation with Fluoroscopy (Category 1)

	Fluoroless Ablation (Category 1)	Traditional Ablation with Fluoroscopy (Category 1)	p-value
Category details	Ablation with both CWPM and ICE	Ablation with visual occulusion	
No. of freezes	213	187	
Temp at 30 seconds	−31.7°C ± 5.9°C	−32.8°C ± 4.7°C	0.0373
Minimum temperature	−47.4°C ± 6.3°C	−47.7°C ± 9.2°C	0.677
Thaw time to 20°C	50.4 ± 30.6 seconds	50.0 ± 32.7 seconds	0.116
Time to Isolation	56.2 ± 28.1 seconds	46.8 ± 32.0 seconds	0.2121
Procedure time	102.2 ± 27.3 minutes	104.5 ± 16.9 minutes	0.6436
Fluoroscopy time	0.00 minutes	17.0 ± 7.4 minutes	< 0.0001

CWPM: continuous wave pressure monitoring; ICE: intracardiac echo.
